# Ablation of a life-threatening arrhythmia in a patient with Brugada syndrome

**DOI:** 10.21542/gcsp.2021.4

**Published:** 2021-04-30

**Authors:** Omnia Kamel, Wessam Gamal, Aliaa Tarek, Walaa Ibrahim, Mohamed Sayed, Josep Brugada, Javier Moreno

**Affiliations:** 1 Aswan Heart Centre, Aswan, Egypt; 2 Hospital Clinic, University of Barcelona, Spain; 3 Hospital Ramon y Cajal, Madrid, Spain

## Abstract

Brugada syndrome is an autosomal dominant arrhythmogenic disease associated with an increased risk of ventricular fibrillation and sudden cardiac death. The mainstay of treatment in high-risk patients is an implantable cardioverter-defibrillator (ICD), however radiofrequency ablation has been proposed over the past decade as an additional therapy in patients with recurrent ICD firing. We report a case of Brugada syndrome with an electrical storm which was successfully managed by radiofrequency ablation.

## Introduction

**Figure 1.**
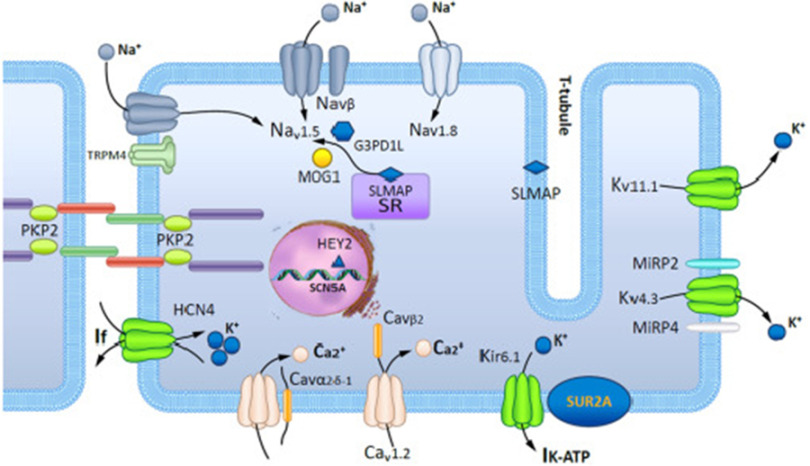
Schematic representation of a cardiomyocyte exhibiting the proteins involved in Brugada syndrome pathogenesis.

First described in 1992, Brugada syndrome (BrS) is an inherited condition characterized by ST-segment elevation in the right precordial leads, associated with ventricular arrhythmias (VAs) and sudden cardiac death (SCD), especially in young males^1^. Loss of function in the SCN5A gene, which controls voltage gated sodium channels, is found in approximately 30% of affected individuals (Figure 1)^2^.

The standard therapy for the prevention of SCD in BrS is the use of an implantable cardioverter-defibrillator (ICD) especially in patients who have experienced a prior cardiac arrest or sustained VAs. However, ICDs do not prevent the occurrence of VAs but react to terminate/defibrillate the arrhythmic episode, thereby preventing SCD.

Quinidine has been reported to reduce the ventricular arrhythmic burden in BrS, however, quinidine is not widely available and has been associated with intolerable adverse effects^3^. In the last decade, substrate ablation of the right ventricular outflow tract (RVOT)/right ventricle (RV) has emerged as a promising therapeutic option for BrS patients with recurrent VAs.

## Case report

**Figure 2.**
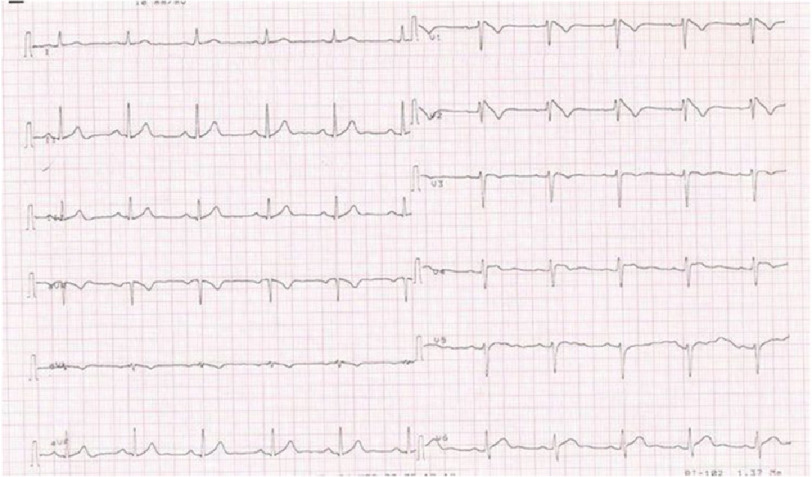
High precordial leads ECG showing Brugada pattern.

**Figure 3.**
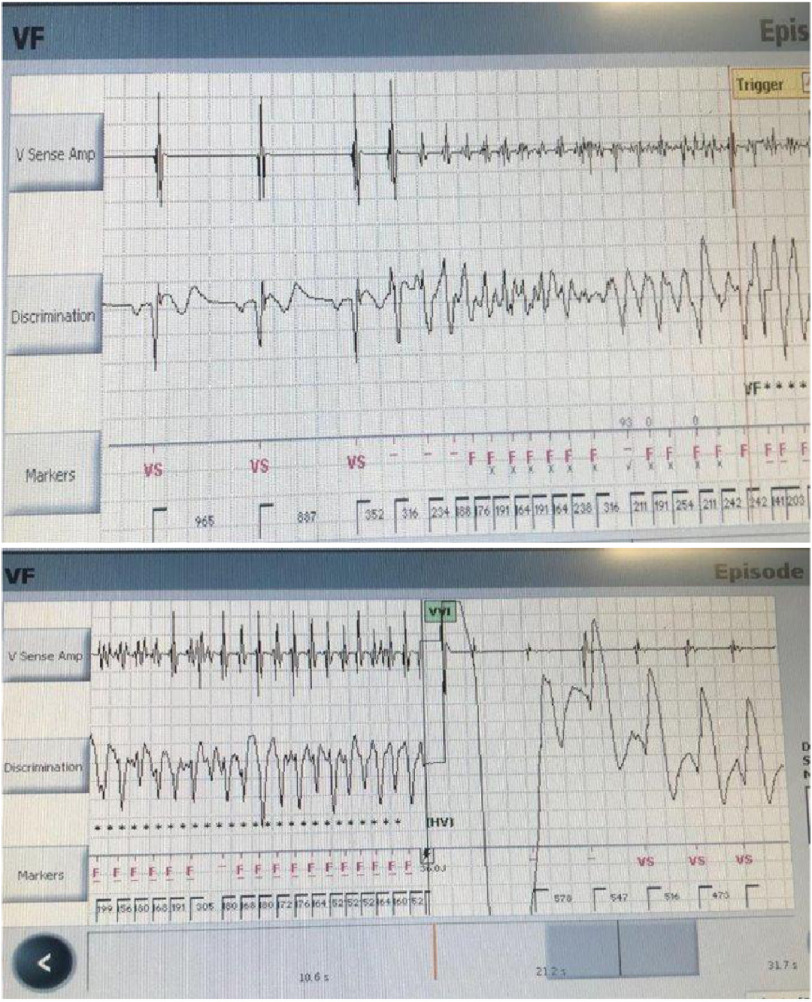
(A, B). Interrogation of the ICD showing an episode of VF successfully terminated by internal shock.

We report the case of a 28-year-old male patient who was discovered to have Brugada pattern on his surface ECG, after resuscitation from sudden cardiac arrest (Figure 2). He fully recovered and had an ICD implantation before hospital discharge. Three months later, he had a ventricular fibrillation (VF) storm with repeated ICD shocks (Figures 3A, 3B). He underwent an epicardial ablation attempt using non-high-density 3D mapping, which targeted substrate modification at anterior RVOT. However, two months later, he had another VF storm. The patient presented to our Centre for further management and a repeat ablation was deemed appropriate.

**Figure 4.**
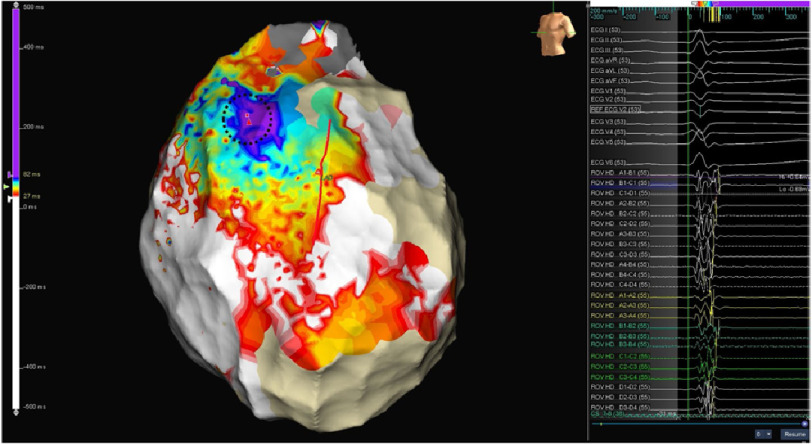
Baseline epicardial activation map in sinus rhythm before flecainide infusion. LAO view. Purple area shows baseline latest recorded depolarization electrograms, fragmented and with multiple components. Straight red line delineates left anterior descending artery location.

**Figure 5A.**
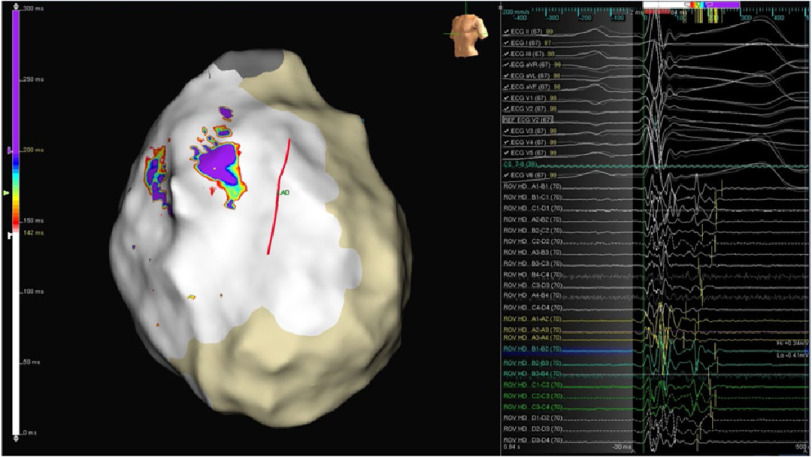
Epicardial activation map after flecainide infusion, with a new color scale to highlight areas with extended fragmentation. Purple areas show the new latest recorded electrograms, much later than before, and also fragmented and with multiple components.

**Figure 5B.**
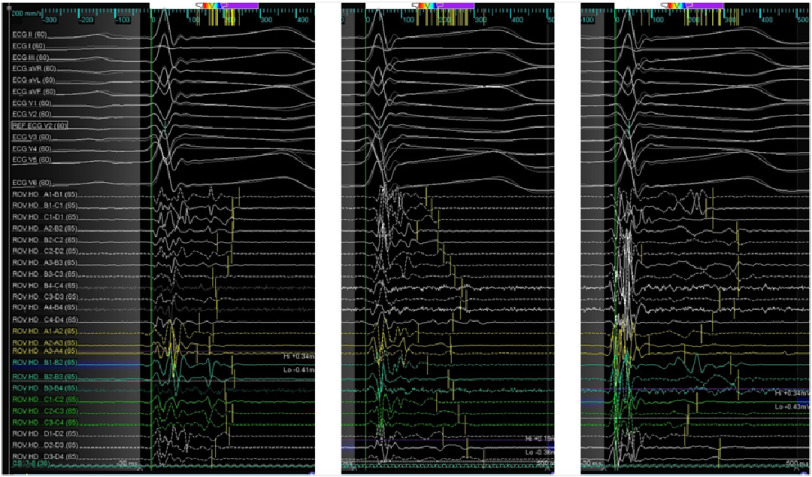
Local electrograms at abnormal areas highlighted in purple in previous figure. Multiple repolarization abnormalities are shown spanning up to the peak of the ECG T wave. Latest deflections are marked by yellow bars.

**Figure 6.**
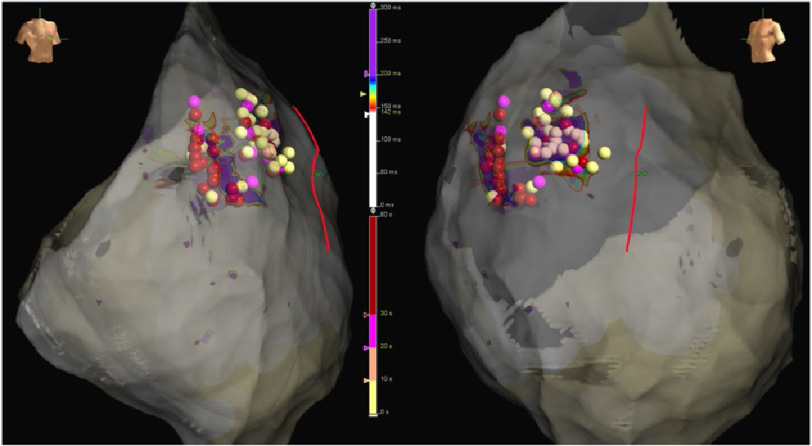
RAO and LAO views of the ablated areas. Ablation dots are color-coded according to radiofrequency duration. Longest applications, over 30 seconds, are colored in red.

**Figure 7.**
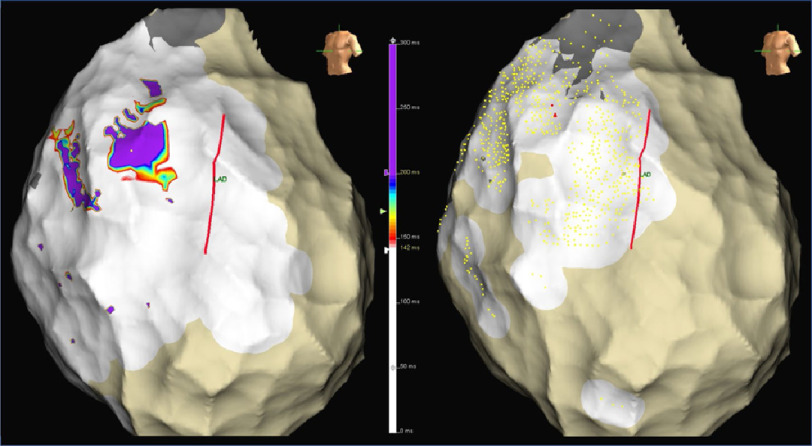
Epicardial activation maps on flecainide before (left) and after ablation (right) using same time scale. LAO views. No late activated purple areas are identified anymore after thorough local ablation.

**Figure 8.**
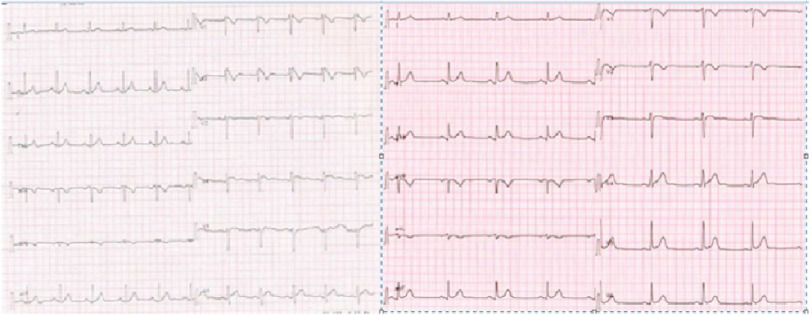
Surface ECG before (on the left) and after the successful ablation (on the right) with disappearance of the manifest Brugada pattern.

After informed consent, the procedure was performed under general anesthesia. Two right femoral venous accesses were obtained, one for positioning of CS catheter, the other for endocardial mapping of RV. A Chiba spinal needle was used for the epicardial access. To avoid epicardial coronary artery injury, a coronary angiography was performed to visualize the course of the left anterior descending artery (LAD). High-density substrate mapping was performed on RV endocardium and epicardium using an Advisor HD grid mapping catheter and Ensite Precision mapping system (Abbott Medical, Minnesota, USA).

In sinus rhythm we looked for abnormal electrograms (EGMs) which included fractionated potentials, split EGMs, isolated late potentials and low voltage EGMs (Figure 4). These abnormal EGMs were tagged before and right after flecainide infusion (2 mg per kg in 10 minutes). The interesting abnormal signals were found on this second procedure only on a limited area at the epicardial surface of the anterior RVOT (Figures 5A, 5B). Therefore, we ablated only the sites of these abnormal EGMs using TactiCath™ Contact Force Ablation Catheter, Sensor Enabled™ at 35–45 W (Figure 6). After ablation, a remap showed that the pre-determined endpoint, abolition of all abnormal electrocardiograms, was accomplished as shown after flecainide infusion (Figure 7).

The patient recovered well after the procedure and was discharged home the next day. After one year of close follow up, there have been no recurrences of ventricular arrhythmias upon frequent interrogations of his ICD (Figure 8).

## Discussion

Catheter ablation for BrS patients with recurrent VF was first introduced by Haïssaguerre *et al* in 2003, where they targeted premature ventricular complexes (PVCs) originating from RVOT that were thought to trigger VF. Given the fact that PVCs in patients with BrS could be quite scarce, wide clinical application of this idea was impractical^4^.

In 2009, an experimental study on mongrel dogs with induced Brugada pattern concluded that radiofrequency catheter ablation applied to the epicardium of the RVOT may be more effective than endocardium in elimination of VAs^5^. Over the following years, there was growing evidence that epicardial substrate modification is beneficial in abolishing the recurrence of VF. Most importantly, in 2011, Nademanee *et al* reported normalization of the ECG pattern and abolition of recurrent VF episodes after epicardial RVOT substrate ablation in a cohort of 9 patients with Brs and recurrent ICD shocks^6^.

In 2017 multicenter trial, Pappone, Brugada, Santinelli and colleagues published the results of epicardial ablation in 135 consecutive patients. In this large series, the study group determined the arrhythmogenic electrophysiological substrate before and after ajmaline provocation using three-dimensional electroanatomical mapping. Elimination of this substrate led to subsequent ECG normalization and VT/VF non-inducibility in all ablated patients. More importantly, the group reported no complications related to the picture^6^. Recently, Talib *et al*, in a series of 21 patients, introduced an endocardial approach as a first step in ablation of patients with BrS with an overall success rate of 67% ^8^.

As our case was a redo case, it was reasonable to map both endocardial and epicardial surfaces of the RV using high-density mapping technology to ensure full coverage of all possible electrical substrates. However, all significant signals were present only on the epicardial surface. Thorough high-density mapping, after sodium channel blocker infusion, clearly identified areas of abnormal EGMS that were possibly missed at the first attempt, or most likely, not sufficiently ablated.

It is important to highlight that despite the invasive nature of epicardial mapping and ablation, studies on this approach for patients with BrS and other conditions, such as arrhythmogenic right ventricular cardiomyopathy and dilated cardiomyopathy, consistently showed low periprocedural complication rates. Careful handling of the puncture of the epicardial space with adequate experience and knowledge of fluoroscopic anatomy of RV, renders this approach mostly safe^7^.

### Learning points

• Catheter ablation is a valid option in patients with BrS who experienced recurrent shocks.
• The use of sodium channel blockers helps to unmask the specific electric substrate in BrS.
• The appropriate use of up-to-date technologies, such as high-density mapping, can significantly improve the outcomes in complex ablation procedures.
